# The Impact of Matching to Psychotherapy Preference on Engagement in a Randomized Controlled Trial for Patients With Advanced Cancer

**DOI:** 10.3389/fpsyg.2021.637519

**Published:** 2021-02-24

**Authors:** Allison Marziliano, Allison Applebaum, Anne Moyer, Hayley Pessin, Barry Rosenfeld, William Breitbart

**Affiliations:** ^1^Department of Medicine, Center for Health Innovations and Outcomes Research, Northwell Health, New York, NY, United States; ^2^Department of Psychiatry and Behavioral Sciences, Memorial Sloan Kettering Cancer Center, New York, NY, United States; ^3^Department of Psychology, Stony Brook University, New York, NY, United States; ^4^Department of Psychology, Fordham University, New York, NY, United States

**Keywords:** patient preferences, engagement, matching, attrition, alliance

## Abstract

**Objective:** This study examined whether patients who were randomly assigned to their preferred therapy arm had stronger engagement with their treatment than those who were randomly assigned to a non-preferred therapy arm.

**Method:** Data were drawn from a RCT comparing Individual Meaning-Centered Psychotherapy (IMCP), with Individual Supportive Psychotherapy (ISP), in patients with advanced cancer. Treatment engagement was operationalized as patients' perceptions of the therapeutic alliance with their therapist and therapy sessions attended. Two 2 by 2 Analysis of Variance (ANOVA) models were used, with treatment preference (IMCP vs. ISP) and treatment assignment (IMCP vs. ISP) as the independent variables and working alliance and number of sessions attended as outcome variables.

**Results:** Patients who preferred and were assigned to IMCP reported a significantly stronger alliance than those who preferred IMCP but were assigned to ISP.

**Conclusions:** The findings from this study have broader implications for research on psychotherapy beyond the appeal of IMCP in advanced cancer patients. Patients who prefer a novel psychotherapy that they cannot engage in elsewhere, but receive the standard treatment may experience weaker alliance than patients who prefer the standard but receive the novel therapy.

**Trial registration:** Clinicaltrial.gov ID: NCT01323309

## Introduction

The key component of a randomized controlled trial (RCT) is randomization, whereby study participants are randomly assigned to different treatment conditions as a means of balancing potentially confounding variables across groups (Sacks et al., 1982; Pocock, 1983; Chambless and Hollon, 1998; Lohr and Carey, 1999). Although not typically the focus of RCTs, this study design also provides the opportunity to investigate the dynamics of randomly assigning patients to treatments which they do or do not have a preference for. In the context of psychotherapy research, this process allows for an analysis of the effect of treatment preferences on the treatment process and outcome.

One key component of the therapeutic process is the extent to which a patient (or client) is engaged in treatment. Treatment engagement can be measured in multiple ways, ranging from a simple dichotomous variable reflecting treatment completion vs. premature termination (i.e., attrition) to the therapeutic alliance between patient and therapist (Greenson, 1965). Indeed, measures of therapeutic alliance are commonly used in many types of psychotherapy outcome research, whether as a dependent variable or as a covariate. One variable that may impact treatment engagement in the context of RCTs of psychotherapy interventions is the extent to which a patient receives their preferred treatment approach (i.e., they are “matched” to their preferred treatment).

Much of the literature on treatment preferences in RCTs has focused on attrition, with considerable evidence suggesting that patients who are assigned to their preferred treatment arm (matched) in psychotherapy trials are more likely to remain in the study than those assigned to an arm they did not prefer. In a meta-analysis examining impact of preference on outcomes in various mental disorders, Swift and Callahan (2009) found that patients who were matched to their preferred psychotherapy were 50% less likely to drop out of the study than those patients who were mismatched. They suggested that patients may resent not receiving their preferred treatment, thereby leading to a more negative attitude toward the treatment and/or RCT or disappointment more generally. They cited a construct termed “resentful demoralization” (Bradley, 1996; Bowling and Rowe, 2005) as justifying the greater risk of study attrition across conditions. These studies notwithstanding, other research on attrition has demonstrated little impact of treatment matching on attrition (King et al., 2005; Floyd and Moyer, 2010). For example, in a RCT comparing a group-based intervention (Meaning-Centered Psychotherapy) to a supportive group psychotherapy, the authors found no evidence that participants matched to their preferred treatment were more likely to drop out of the study compared to those who were mismatched to their preferred treatment (Applebaum et al., 2012b).

To date very little research has analyzed the impact of treatment matching on therapeutic alliance more generally. One such study demonstrated that in a sample of patients with Major Depressive Disorder, mismatch between preferred and randomized treatment corresponded to a weaker working alliance during the treatment (Kwan et al., 2010). In another study of 75 patients with Major Depressive Disorder enrolled in an RCT comparing supportive expressive therapy, sertraline, and placebo (the latter two of which also received weekly clinical management sessions with a pharmacotherapist), patients who preferred and were matched to psychotherapy had a stronger working alliance with their therapists than patients who preferred psychotherapy but were assigned to one of the other treatment arms (Iacoviello et al., 2007). This limited research, while supporting a hypothesized impact of preference matching in RCTs, is complicated by the question of whether treatments are equally desirable.

The present study examined whether patients who were randomly assigned to their preferred psychotherapy arm (i.e., matched) in a RCT of alternative psychotherapy approaches for advanced cancer patients had greater engagement with the assigned treatment (defined as a stronger working alliance with the therapist and a greater number of therapy sessions attended) than those who were assigned to their non-preferred therapy arm (mismatched). Based on the literature reviewed above, we hypothesized that patients matched to their preferred treatment would have a stronger therapeutic alliance with their therapist than those mismatched to their preferred treatment. In addition, we hypothesized that there would be no difference in number of psychotherapy sessions attended between those who were matched and mismatched to their preferred treatment. Although the existing literature in this area shows inconsistent findings, our hypothesis is based on the results from the RCT of the group version of IMCP, in which Applebaum et al. (2012b) found that matching to preference did not impact attrition rate.

## Materials and Methods

The data come from a larger trial (for additional Methods, see Breitbart et al., 2018) that examined the efficacy of a novel type of therapy, Individual Meaning Centered Psychotherapy (IMCP), compared to a standard form of supportive psychotherapy (Individual Supportive Psychotherapy or ISP), and a group receiving enhanced usual care (EUC; excluded from these analyses), in improving psychological and existential distress in advanced cancer patients. Outcome measures for the larger study included improved sense of meaning in life, spiritual well-being, and overall quality of life, and reduced psychological distress including depression, anxiety, hopelessness, and desire for hastened death. The focus of the analyses for this manuscript was to compare the effects of patient match/mismatch to their preference for the type of psychotherapy intervention (IMCP or ISP) on treatment engagement. For our study, treatment engagement was operationalized as patients' perceptions of the therapeutic alliance with their therapist and therapy sessions attended. We defined treatment engagement in this way based on existing literature, which suggests that alliance and session attendance represent facets of treatment engagement (Thompson et al., 2007; Loveland and Driscoll, 2014).

Criteria for inclusion in the larger study were: (1) age 21 years or older; (2) score of four or higher on the Distress Thermometer (Roth et al., 1998); (3) Karnofsky Performance Rating Scale (Karnofsky and Buchenal, 1949; Schag et al., 1984) score of 60 or greater; (4) ability to understand and communicate in English; and (5) a confirmed diagnosis of stage 4 cancer of the breast, prostate, colon, or solid tumor malignancies, locally recurrent ovarian cancer, or confirmation from the treating physician and documentation in the research medical record of advanced disease. Patients were excluded if they had a severe psychiatric disturbance as determined by the research study assistant or physical limitations sufficient to preclude participation.

Between March 2011 and March 2016, 6,410 patients who met the inclusion criteria were approached for possible study participation either in person during their chemotherapy treatment, by telephone or informational mailing. At this point, patients were given adequate information and education about each of the three possible treatment arms in this RCT so they would be able to state an informed preference. In addition, patients were provided with a copy of the consent form to review, which included details on each of the treatments. The description of IMCP in the consent form included the following language: IMCP will focus on how to maintain or even increase a sense of meaning and purpose in life; each session has a specific topic such as what is meaningful to you and how cancer has changed this; and written exercises, homework, and a larger project. The description of ISP in the consent form included the following language: helping you cope with cancer by giving you a place to express your feelings and get support. You will be asked to share your concerns and discuss how you are feeling about these issues. Lastly, the description of EUC in the consent form included the following language: referrals based on your individual needs; additional written material with information on how to cope better when you have cancer and additional resources that may be helpful. Patients who declined to participate cited various reasons such as limited time, lack of interest, or geographic or scheduling barriers.

A total of 321 patients agreed to participate, and completed the consent form and a battery of pre-randomization measures. Shortly thereafter, participants were randomized to one of the three treatment arms: IMCP, ISP or EUC. Those receiving either of the two psychotherapy treatments received seven 1-hour individual psychotherapy sessions. All study therapists had at least a Master's degree in a mental health discipline (e.g., psychology, psychiatry or social work), and were trained and supervised by a licensed, experienced doctoral-level clinician. Psychotherapy sessions were audio recorded for review during supervision, as well as to assess treatment integrity. To protect against cross-contamination effects, different study therapists were used for each psychotherapy arm. Participants were offered a $20 travel reimbursement for each session. Patients in either of the two treatment arms completed a battery of questionnaires at four different time points: immediately before the first therapy session/baseline (T1), immediately before the fourth therapy session/midpoint (T2), directly following the last session/post-intervention (T3), and 8–12 weeks following completion of the T3/follow-up (T4). Demographic information, health-related information, and participant preferences for the type of therapy received were elicited prior to randomization, at the time participants provided informed consent. All participants in this research provided written consent to the inclusion of material pertaining to themselves, acknowledge that they cannot be identified via this manuscript and understand that they are fully anonymized. This study was approved by the Institutional Review Boards of Memorial Sloan Kettering Cancer Center and Stony Brook University. The clinical trial registration number for the parent RCT is NCT01323309.

### Measures

Pre-randomization Preference Questionnaire (PRP). This 4-item questionnaire was developed by the investigators to assess patients' preferences for aspects of the three types of psychotherapy interventions. This measure consists of three questions soliciting to what degree (not at all, slightly, somewhat, quite a bit, very much) participants prefer the program to focus on (1) providing support, (2) talking about feelings about cancer, and (3) finding a sense of meaning and purpose in life despite having cancer. The fourth question asks participants to indicate their preference, if they have one, from four options: IMCP, ISP, EUC, and no preference. Participants were informed that their responses were solely for analytical purposes and would not influence their therapy assignment. For the purposes of these analyses, groups were defined based on the preferences reported in the fourth question only. This measure was administered at T1/baseline only.

Working Alliance Inventory-Short Form (WAI-SF). The original WAI is a 36-item instrument designed to measure variables affecting the degree of counseling success independent of the therapist's theoretical orientation (Horvath and Greenberg, 1989). There are three subscales of the WAI (Tasks, Goals and Bond) as well as a composite score. In the original validation study, Cronbach's coefficient alpha for the WAI composite score was 0.93 (Horvath and Greenberg, 1986). This study utilized a shortened version of the WAI, which consists of a 12-item total score, and four items in each of the three subscales. Validity has been demonstrated for the WAI-SF based on a similar factor structure with the original 36 item WAI. Cronbach's alpha for the WAI-SF total is 0.98, and for each subscale, is 0.90, 0.92, and 0.90 for the task, bond, and goal factors, respectively (Tracey and Kokotovic, 1989). This measure was administered at T2 only (midway through the interventions) and results reported in this manuscript are drawn from this assessment.

### Analytic Strategy

To investigate whether being assigned to one's preferred treatment arm impacted treatment engagement, two 2 by 2 Analysis of Variance (ANOVA) models were used, with treatment preference (IMCP vs. ISP) and treatment assignment (IMCP vs. ISP) as the independent variables and level of working alliance and number of sessions attended as outcome variables. The statistical software program, G^*^power (Faul et al., 2007), was used to estimate the sample size necessary to conduct each of the two 2x2 ANOVA analyses with 80% power and a medium effect size of *f* = 0.25. The program yielded a required sample size of 128 participants. Data were analyzed using Statistical Package for the Social Sciences (SPSS), Version 21.

## Results

The study sample (*N* = 254) was mostly female (*n* = 185; 72.8%), White (*n* = 205; 80.7%), non-Hispanic (*n* = 228; 89.8%), and married (*n* = 157; 61.8%). More than half of the sample identified as either Catholic (*n* = 82; 32.3%) or Jewish (*n* = 68; 26.8%) (see Table 1 for additional demographic data). The average age of the sample was 57.65 (*SD* = 10.94), ranging from 25 to 85 years old. The average years of education completed by this sample was 16.59 (*SD* = 2.57), with a range of 10–25 years of education. The mean score on the WAI-SF was 68.96 (*SD* = 11.87, range 27–84) and the mean number of sessions attended was 5.75 (*SD* = 2.86, possible range 0–7).

**Table 1:** Descriptive statistics (*N* = 254).

**Category**	**Frequency**	**Percent**
**GENDER**		
Male	69	27.20%
Female	185	72.80%
**RACE**		
Caucasian	205	80.70%
African Am.	24	9.40%
Asian	11	4.30%
Other	14	5.50%
**ETHNICITY**		
Non-Hispanic	228	89.80%
Hispanic	24	9.40%
Unknown	2	0.80%
**MARITAL STATUS**		
Single	37	14.60%
Married	157	61.80%
Widowed	18	7.10%
Separated	8	3.10%
Divorced	29	11.40%
Cohabitating	5	2.00%
**RELIGION**		
Catholic	82	32.30%
Protestant	14	5.50%
Jewish	68	26.80%
Baptist	3	1.20%
Muslim	3	1.20%
Other	44	17.30%
None	40	15.70%

When asked to indicate their preference for IMCP, ISP, or EUC, 141 (43.9%) endorsed IMCP, 113 (35.2%) patients endorsed ISP, 13 (4.0%) patients endorsed EUC, and 54 (16.8%) patients indicated “no preference.” Patients who endorsed EUC or “no preference” were excluded from subsequent analyses, as were patients randomly assigned to EUC (regardless of preference). Of the 254 participants who indicated IMCP or ISP as their preference, 116 were assigned to either IMCP or ISP, and completed the WAI-SF at T2, mid-way through treatment.

**Figure 1:**
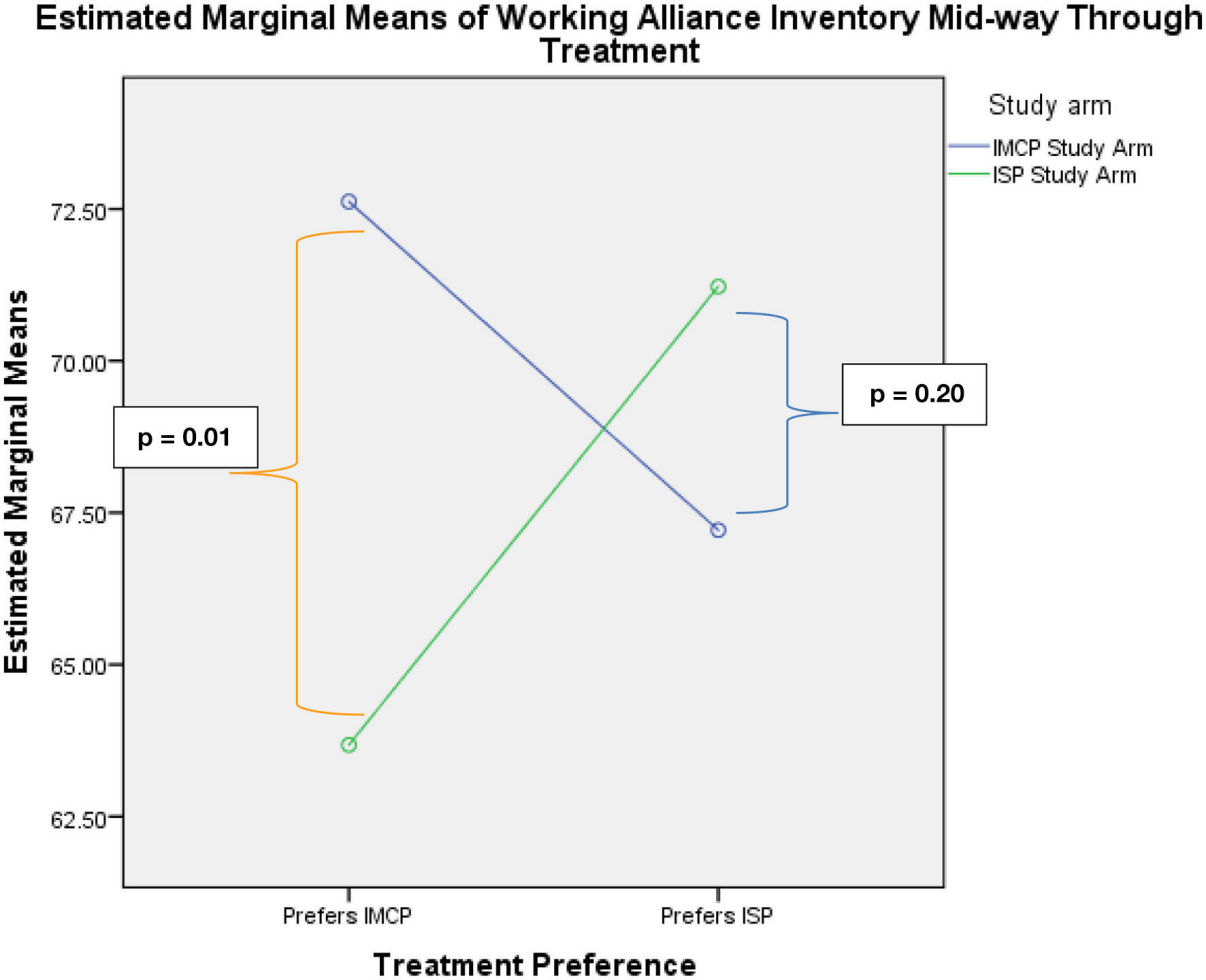
Significant crossover interaction and pairwise comparisons indicating a significant difference between patients who prefer and receive IMCP and those who prefer IMCP and receive ISP.

As hypothesized, the ANOVA model predicting WAI-SF total score from treatment preference and treatment assignment generated a significant interaction effect, *F*(1, 116) = 9.41, *p* = 0.003. Simple effects analyses (see Figure 1) demonstrated that patients who preferred IMCP and were assigned to IMCP indicated the strongest treatment alliance (*M* = 72.62, *SD* = 10.83), whereas patients who preferred IMCP and were assigned to ISP had the weakest treatment alliance (*M* = 63.68, *SD* = 10.58), and this difference was statistically significant, *F*(1, 116) = 9.69, *p* < 0.01, *d* = 0.83, indicating a large effect size. By contrast, in patients who preferred ISP, there was no significant difference in the mean treatment alliance between those assigned to IMCP (*M* = 67.21, *SD* = 14.61) and those assigned to ISP (*M* = 71.22, *SD* = 9.31), *F*(1, 116) = 1.68, *p* = 0.20, although the effect size was medium, *d* = −0.33. There was no significant main effect for treatment preference in this model, *F*(1, 116) = 0.26, *p* = 0.61, *d* = −0.09, indicating a very small effect size and no significant difference on the WAI-SF between those who preferred IMCP (*M* = 68.15, *SD* = 11.59) and those who preferred ISP (*M* = 69.22, *SD* = 11.47). Likewise, there was no significant main effect for treatment assignment, *F*(1, 116) = 1.37, *p* = 0.25, *d* = 0.21, indicating a small effect size and no significant difference on the WAI-SF between those who were assigned to IMCP (*M* = 69.92, *SD* = 11.59) and those assigned to ISP (*M* = 67.45, *SD* =11.47).

A larger subset of the 254 participants who expressed a preference for IMCP or ISP were included in the analysis of treatment match on number of sessions attended (*n* = 163). This ANOVA model did not generate a significant interaction between treatment preference and treatment assignment, *F*(1, 163) = 1.60, *p* = 0.21. Once again, there was no significant main effect for treatment preference, indicating no significant difference in number of sessions attended between those who preferred IMCP (*M* = 4.91, *SD* = 2.93) and those who preferred ISP (*M* = 5.39, *SD* = 2.94; *F*(1, 163) = 1.09, *p* = 0.30, and a small effect size, *d* = −0.16). There was also no significant main effect for treatment assignment, *F*(1, 163) = 0.82, *p* = 0.37, *d* = 0.14, indicating no significant difference in number of sessions attended between those who were assigned to IMCP (*M* = 5.36, *SD* = 2.99) and those assigned to ISP (*M* = 4.94, *SD* = 2.96) and a small effect size.

## Discussion

This study represents one of very few analyses of the impact of treatment preferences on therapeutic alliance in an RCT, and the first to focus on patients with advanced cancer, for whom development and evaluation of novel treatments is a primary concern. As hypothesized, the results demonstrated a significant impact of treatment matching on therapeutic alliance, but this effect was only present for those participants who preferred IMCP, not for those who preferred ISP. Moreover, as hypothesized, this effect was only evident on the Working Alliance Inventory, not on a more simplistic measure of treatment engagement based on number of sessions attended. This pattern of results is likely to reflect several factors, including the greater variability and sensitivity of the WAI-SF compared to number of sessions attended, and the important differences between the two treatment approaches.

Although the proportion of patients who expressed a preference for IMCP was roughly comparable to the proportion that preferred ISP, these findings are likely a reflection of the greater appeal of meaning-based interventions, particularly for patients with advanced cancer. Moreover, while it is likely that patients in IMCP found the treatment no less “supportive” than the ISP intervention, the converse is not likely to be true; patients in ISP would not perceive the intervention as focusing on themes of personal meaning or purpose in life. In addition, IMCP is a novel technique that is not routinely offered to patients, whereas supportive psychotherapy approaches are commonly used in a wide range of settings. Thus, patients with a preference for IMCP may have felt greater disappointment when receiving ISP because they had “missed” the opportunity to engage in this treatment, and this disappointment may have colored their relationship with the therapist. Of note, the measure of therapeutic alliance used for this study, the WAI-SF, ranges from 12 to 84. Thus, although alliance was stronger for some patients and groups, it was generally strong for the sample overall.

The absence of any impact of preference matching on the number of sessions attended was also hypothesized based on the findings from Applebaum et al. (2012b), that matching to preference did not impact attrition. Moreover, there are clear limitations to reliance on number of sessions attended as an indicator of treatment engagement, as attendance does not capture the extent to which participants were engaged in the session, completed homework assignments on time, or completed the sessions within the expected time frame. For example, unenthusiastic participants may miss and/or reschedule sessions due to a lack of enthusiasm, but ultimately complete all or most of the allotted sessions. On the other hand, enthusiastic participants might miss sessions due to circumstances beyond their control (i.e., progressive illness, surgery, physician appointments). Given the limited range in this variable (which can only range from 0 to 7), it is not surprising that weaker, non-significant effects were observed for attendance whereas significant effects were found for a presumably more sensitive variable of therapeutic alliance.

As noted, our measure of therapeutic alliance was administered at one time point only: midway through the course of the intervention. Our reasons for selecting midway through the course of the intervention as an appropriate timepoint at which to measure therapeutic alliance are three-fold: (1) we strived to balance the number of measures administered across different timepoints so as not to burden our patients, as filling out questionnaires could be quite cumbersome and could take up to 1-hour in some cases; (2) we felt that the Working Alliance Inventory was an appropriate measure to be administered at mid-point of the intervention course (rather than post-intervention) because some research indicates that many facets of the perception of therapeutic alliance stabilize by the 5th session. In one study in the literature (Bachelor and Salame, 2000), alliance perceptions were for the most part stable from the 5th to 10th therapy sessions; (3) there appears to be a stronger association between measures of therapeutic alliance and treatment outcome when alliance is measured at earlier stages of treatment (Elvins and Green, 2008). Although patients' psychological outcomes were not the focus of this manuscript, we were interested in exploring those, as well; and (4) we did not want therapeutic alliance to be confounded by termination of therapy.

### Limitations

There are several limitations inherent in studying patient preferences for treatment. First, it is difficult to define and identify the extent of a participant's preference, particularly when limited information is provided as to the precise nature of the intervention (which is inevitable in psychotherapy outcome research). A systematic review (King et al., 2005) of the impact of patient and physician preferences for interventions in RCTs concluded that patients' immediate responses may change upon deeper reflection in the minutes, hours, and days after a preference is indicated. Preferences may also be influenced by framing effects, verbal descriptions of risks and benefits, and the preference or characteristics of the individual presenting the possible treatments (Edwards and Elwyn, 2001; Jenkins et al., 2001). It is unclear, due to a lack of research on the topic, how anticipated preferences for treatment, perceptions of the actual treatment and post-treatment preferences relate to each other (King et al., 2005). Second, although patients engaging in RCTs may express treatment preferences, they have all agreed to randomization; thus, their preferences are likely weak. This limitation, in particular, may explain any non-significant findings. On a related note, we did not include any post-randomization measures of treatment aversion, for example, to determine whether patients were actually assigned to a non-preferred treatment, nor did we assess potential confounding variables or whether treatments were considered equally desirable.

In regard to the sample more generally, it should be noted that the study participants comprised a fairly homogenous group of highly educated, employed, and Caucasian patients. Thus, generalizability to other, more ethnically and socioeconomically diverse populations is unknown. Further, the institution at which this study was conducted has a culture where patients often have preferences for the novel therapy, rather than the standard of care, indicating a potential bias in our sample. Similarly, as with any RCT, these study findings only include the subset of individuals who agree to be randomized, again limiting the generalizability to the broader population of cancer patients. It should also be noted that study therapists were aware of patient preference at the start of treatment and could have worked harder to establish an alliance if there was a mismatch (diminishing the impact of a preference mismatch). This is an important limitation, specifically when assessing the preference effect. In addition, all participants received free treatment, which may have mitigated the effect of treatment preference (i.e., because free treatment is often appreciated, even if it is not the treatment that sounds most appealing). Finally, although there is a substantial research literature demonstrating the relationship between therapeutic alliance and treatment outcome (Applebaum et al., 2012a; Sturgiss et al., 2016; Manne et al., 2017), this study did not have sufficient power to analyze whether treatment outcome was mediated by therapy match/mismatch, particularly given that substantial differences in the effectiveness of these treatments has already been demonstrated (Breitbart et al., 2018).

### Conclusion

The findings from this study have broader implications for research on psychotherapy beyond the appeal of IMCP in advanced cancer patients. Matching patients to their preferred treatment appears to impact patients' alliance with their study therapist, which in turn, may impact clinical outcomes. Given that the comparison of IMCP and ISP in this study is analogous to comparing a novel treatment to an established standard of care in other RCTs, it is important to note that alliance, and outcome, may suffer when patients do not receive their preferred treatment when their preference is a novel therapy they cannot engage in elsewhere. This study highlights the importance of educating and actively engaging patients in decisions of what type of intervention they receive to ensure their “buy in” to treatment, particularly if it is novel or more structured psychotherapy, in an effort to strengthen the therapeutic alliance, and ultimately potentially impact the efficacy of treatment.

## Data Availability Statement

The raw data supporting the conclusions of this article will be made available by the authors, without undue reservation, if requested.

## Ethics Statement

This study involving human participants was reviewed and approved by Memorial Sloan Kettering Institutional Review Board. The participants provided their written informed consent to participate in this study.

## Author Contributions

AMa contributed to data cleaning, formulated this study question, conducted analyses, drafted the manuscript, and prepared the manuscript for submission. AA contributed to the study design and conception, provided guidance on the study question, aided with manuscript preparation, and reviewed drafts of the manuscript. AMo directed study analyses, oversaw manuscript preparation, and reviewed drafts of the manuscript. HP contributed to the study design and conception, directed data cleaning, aided with manuscript preparation, and reviewed drafts of the manuscript. BR contributed to the study design and conception, directed analyses, and reviewed drafts of the manuscript. WB contributed to the study design and conception and reviewed drafts of the manuscript. All authors contributed to the article and approved the submitted version.

## Conflict of Interest

The authors declare that the research was conducted in the absence of any commercial or financial relationships that could be construed as a potential conflict of interest.
